# Effect of local endometrial injury in proliferative vs. luteal phase on IVF outcomes in unselected subfertile women undergoing in vitro fertilization

**DOI:** 10.1186/s12958-017-0296-8

**Published:** 2017-09-22

**Authors:** Wenjie Liu, Reshef Tal, He Chao, Minghui Liu, Ying Liu

**Affiliations:** 1 Department of Reproductive Medicine, Beijing Obstetrics and Gynecology Hospital, Capital Medical University, Beijing, 100026 China; 2 Obstetrics, Gynecology & Reproductive Sciences, Yale University School of Medicine, New Haven, CT 06510 USA

**Keywords:** Endometrial injury, Endometrium, In vitro fertilization, Implantation

## Abstract

**Background:**

Mechanical endometrial injury prior to IVF has been suggested as a means to increase implantation rates by improving endometrial receptivity. However, the effects of endometrial injury in proliferative vs. luteal phase have not been studied before. This study aimed to explore whether endometrial injury in the proliferative phase of the preceding cycle before in vitro fertilization/embryo transfer (IVF-ET) improves the clinical outcomes in unselected subfertile women compared with injury in luteal phase.

**Methods:**

A group of 142 patients who were good responders to hormonal stimulation were randomized into four groups: injury group (group A: endometrial injury in proliferative phase, *n* = 38; group B: endometrium injury in luteal phase, *n* = 32), and non-injury group as control (group C: non-injury in proliferative phase, *n* = 36; group D: non-injury in luteal phase, *n* = 36). Patients in injury groups underwent endometrial injury in either proliferative phase or luteal phase in the preceding cycle before IVF treatment. Clinical outcomes including implantation, pregnancy, and live birth rates were analyzed among the four groups.

**Results:**

The baseline characteristics of the four groups including age, body mass index, duration, type and causes of infertility were similar. There were no significant differences in implantation, clinical pregnancy or live birth rates between injury group and non-injury group. Moreover, there were also no significant differences in implantation, clinical pregnancy, or live birth rates in injury in proliferative phase compared with luteal phase.

**Conclusions:**

Endometrial injury in the cycle preceding IVF of unselected subfertile women does not increase implantation, clinical pregnancy, or live birth rates. Furthermore, there is no significant difference in clinical outcomes between endometrial injury in the proliferative phase and injury in the luteal phase.

**Trial registration:**

This study was retrospectively registered on May 26th, 2017 (ChiCTR-IOR-17011506).

## Background

In vitro fertilization is the last solution for many infertility patients. Although much progress has been achieved in reproductive medicine to improve embryo culture, selection and transfer techniques, the live birthrate per embryo transfer following IVF is still relatively low (<40%) [1]. Implantation failure is still one of the major factors limiting the success of IVF [2]. Embryo implantation is a complex process requiring a precise crosstalk between the embryo and endometrium [3]. A successful implantation mainly depends on two basic factors including embryo quality and endometrial receptivity [4]. Methods to improve IVF outcome are geared towards improving embryo quality/selection and endometrial receptivity. Despite various strategies used to improve the embryonic factors, such as optimization of culture media, assisted hatching, blastocyst transfer, and preimplantation genetic screening (PGS), embryo implantation still commonly fails [5]. The implantation failure may occur during IVF even though the quality of embryos is high, suggesting that the endometrium plays an important role in successful implantation.

Endometrium is a complex dynamic tissue comprised of two zones, the basalis and functionalis layers, undergoing a series of morphological and biochemical changes in each menstrual cycle. During the implantation window, the embryo apposes, attaches and invades the receptive endometrium, which is facilitated by progesterone exposure after sufficient action of estrogen [6]. Furthermore, the endometrium becomes receptive and able to support successful embryo implantation for a limited time, termed the “window of implantation”. In order to improve implantation and pregnancy rates after IVF, it is necessary to develop strategies to optimize endometrial receptivity. However, only few interventions have been attempted to overcome suboptimal endometrial receptivity, including hormonal treatment, correction of intrauterine anatomic abnormalities and immunotherapy [7, 8].

Mechanical endometrial injury prior to IVF has been suggested as a means to increase implantation rates by improving endometrial receptivity [9–11]. Our previous study has demonstrated that intervention treatment to the endometrial abnormalities can significantly improve endometrial receptivity and improve IVF-ET outcome [12]. Many studies reported on outcomes of endometrial injury in women with recurrent implantation failure (RIF) with conflicting results. While some studies demonstrated that implantation rates, and clinical pregnancy rates improved after endometrial injury [9, 10, 13–17], others showed no benefit [5, 7, 18, 19]. Nevertheless, studies on using endometrial injury in unselected women undergoing IVF are scarce [5]. Furthermore, in the previous studies, endometrial injury was performed in either proliferative phase or luteal phase, or in both proliferative and luteal phase. These two phases are very different and each of them has its own characteristics and function, which may lead to different effects of injury, ultimately leading to different outcomes of IVF.

However, the effects of endometrial injury in proliferative vs. luteal phase have not been studied before. Therefore, the aim of the present study was to determine whether timing of endometrial injury in the preceding cycle before embryo transfer cycle, i.e. proliferative vs. luteal phase, may have a different effect on implantation rates and pregnancy outcomes in unselected subfertile patients undergoing IVF.

## Methods

### Study design and participants

A total of 142 patients, who underwent their first IVF cycle,were selected for this study. The inclusion criteria were: (i) infertile women indicated for IVF treatment; (ii) ≤40 years of age; (iii) a normal uterine cavity demonstrated by saline infusionsonogram; (iiii) basal follicle stimulating hormone (bFSH) <12 IU/L. The exclusion criteria were: (i) endometrium with polyp or fibroid; (ii) hydrosalpinx; (iii) endometriosis. Recruited participants were equally randomized into four groups using a table of random numbers: injury group (group A: endometrial injury in proliferative phase, *n* = 38; group B: endometrial injury in luteal phase, *n* = 32), and non-injury group as control (group C: non-injury in proliferative phase, *n* = 36; group D: non-injury in luteal phase, *n* = 36).

This study was approved by the Institutional ethics committee Review Board of Beijing Obstetrics and Gynecology Hospital, Capital Medical University (KY-2012-138). All participating patients recruited to the study were fully counseled and signed written informed consent.

### Endometrial injury

All patients in proliferative or luteal phase injury groups underwent endometrial injury in the preceding cycle before the scheduled IVF treatment. For patients in proliferative phase group, endometrial injury was performed between cycle day10–12. For patients in luteal phase group, endometrial injury was performed 7–9 days after ovulation. The endometrial injury procedure was performed in a standard approach using a Pipelle catheter (Shanghai Jiabao Medical Healthy Science Company, Shanghai, China). The Pipelle catheter was introduced through the cervix up to the uterine fundus. The piston was drawn back to the end of the sheath to create a negative pressure. The sheath was rotated and moved back and forth within the uterine cavity to ensure adequate endometrial tissue has been obtained. Those patients in control groups had the Pipelle catheter inserted through the cervix but no injury was performed to the endometrium.

### Ovarian stimulation

All patients received the IVF treatment in the subsequent cycle with pituitary down regulation using either the long agonist, short agonist or fixed antagonist protocol. For the long protocol, starting about day 21 of their previous menstrual cycle; for the short protocol, on day 2 of their menstrual cycles, patients were treated with 0.1 mg/day of the Gonadotrophin releasing hormone analogue (GnRH-a) triptorelin acetate (Decapepty1, Ferring GmbH, Kiel, Germany) for pituitary down-regulation and endogenous gonadotrop in depletion. For the antagonist protocol, GnRH antagonist 0.25 mg/day (Cetrotide, Boxter Oncology GmbH, Halle, Germany) was started on the sixth day of stimulation.

For ovarian stimulation, on day 2–3 of the menstrual cycle (baseline), patients underwent transvaginal ultrasound examination and serum estradiol measurement. The patients were started on150–225 IU of recombinant follicle-stimulating hormone (Gonal-F, Merck Serono SA, Aubonne, Switzerland)/hMG (Menopur, Ferring GmbH, Kiel, Germany) daily for stimulation of follicular growth. Ovarian response was monitored by serial transvaginal ultrasound and hormonal monitoring. Further dosage adjustments were based on ovarian response. When one to two leading follicles were ≥18 cm in mean diameter, 250 μ g ovidrel (Merck SeronoS.p.A, Modugno, Italy) was given to trigger final maturation of the oocytes.

### Oocyte Retrieval, Insemination, and Transfer

Oocytes retrieval were scheduled about 36 h after hCG administration under the guidance of transvaginal ultrasound, using a single-lumen 16-gauge needle (Cook, Queensland, Australia).The oocytes were exposed to spermatozoa for insemination after 6 h, and intracytoplasmic sperm injection (ICSI) was used in cases involving male factor infertility. A maximum of two embryos were transferred into the uterus on day 2–3 after oocyte retrieval. Excess good quality embryos were frozen for subsequent transfer.

### Outcome measures

The primary outcome was implantation rate. Secondary outcomes included pregnancy, clinical pregnancy, multiple pregnancy, miscarriage and live birth rates. Clinical pregnancy was defined as the presence of an intrauterine gestational sac on ultrasound at 6 weeks with heart beat. The implantation rate was calculated as the ratio of the number of embryonal sacs detected on ultrasound divided by the number of embryos transferred into the uterus. The biochemical pregnancy rate was defined as the ratio of the number of cycles with a positive hCG per total number of cycles undergoing embryo transfer. The clinical pregnancy rate was expressed as the ratio of the number of cycles with clinical pregnancy divided by the total number of cycles undergoing embryo transfer. The live birth rate was expressed as the ratio of the number of births divided by the total number of cycles undergoing embryos transfer. Outcomes were compared between patients undergoing endometrial injury and non-injury, and between patients undergoing injury in proliferative phase and luteal phase.

### Statistical analysis

The baseline characteristics of the four groups of patients including injury-treated in proliferative phase and luteal phase, and non-injury in proliferative phase and luteal phase groups were compared by ANOVA and chi-square.

The average numbers of embryos transferred were compared using a one-way ANOVA. Comparisons between groups of mode of insemination, biochemical pregnancy, clinical pregnancy, miscarriage rate, ectopic pregnancy, multiple pregnancies and live birth rate were performed by ANOVA and chi-squaretest. The statistical analyses were performed using Excel software (Microsoft Corp., Redmond, WA) and SPSS version 22.0 statistical software (SPSS Inc., Chicago, IL, USA). *P* < 0.05 was considered as statistically significant.

## Results

A total of 142 participants were recruited in this study from February 2012 to November 2014. Participant’s age ranged from 23 to 40 years. Indications for IVF, including tubal, male, unexplained, and mixed causes were similar in these four groups. Baseline characteristics of the four groups including the age, body mass index, duration, type and causes of subfertility are shown in Table 1. There were no significant differences in the baseline characteristics among the four groups (*P* > 0.05).

**Table 1:** Baseline characteristics of patients undergoing endometrial injury in proliferative phase, luteal phase and control groups

Variable	Group A	Group B	Group C	Group D	*P* value
	(*n* = 38)	(*n* = 32)	(*n* = 36)	(*n* = 36)	
Age (y)	31.4 ± 4.0	30.3 ± 3.5	31.7 ± 4.8	31.0 ± 4.0	0.561
BMI (kg/m^2^)	22.15 ± 2.99	21.98 ± 3.11	22.20 ± 3.20	23.68 ± 3.40	0.066
Menstrual cycle length (days)	32.05 ± 6.53	33.28 ± 19.00	31.58 ± 7.62	34.75 ± 25.14	0.388
Duration of subfertility (y)	4.3 ± 3.0	3.3 ± 2.6	3.8 ± 3.0	4.1 ± 2.6	0.213
Type of subfertility					0.101
Primary	19(50.0%)	19(59.4%)	14(38.9%)	24(66.7%)	
Secondary	19 (50.0%)	13(40.6%)	22(61.6%)	12 (33.3%)	
Causes of subfertility					
Tubal	30(78.9%)	25(78.1%)	28(77.8%)	29(80.6%)	0.992
Male	22(57.9%)	17(53.1%)	15(41.7%)	15(41.7%)	0.398
Unexplained	3(7.9%)	1(3.1%)	1(2.8%)	3(8.3%)	0.617
Mixed	13(34.2%)	13(40.6%)	12(33.3%)	11(30.6%)	0.849

*BMI* Body mass index

Date are presented as mean ± SD or n (%)

IVF cycle characteristics are presented in Table 2. With regards to the basal FSH, LH, E2 level, basal AFC and protocols of long agonist, short agonist and antagonist, the distributions of patients in the four groups were not significantly different. There was also no difference in the percentage of cycles using IVF or intracytoplasmic sperm injection (ICSI) among the four groups. Furthermore, there were no significant differences in the cycle stimulation characteristics among the four groups, including the dose and duration of hormonal stimulation, endometrial thickness, peak estradiol, and the number of good-quality of embryos transferred.

**Table 2:** IVF cycles characteristics of patients undergoing endometrial injury in proliferative phase, luteal phase and control groups

Variable	Group A	Group B	Group C	Group D	*P* value
	(*n* = 38)	(*n* = 32)	(*n* = 36)	(*n* = 36)	
Basal FSH(IU/L)	6.85 ± 1.78	6.65 ± 1.76	7.73 ± 1.70	7.17 ± 1.82	0.091
Basal LH(IU/L)	4.29 ± 1.82	4.56 ± 1.83	4.34 ± 2.07	5.05 ± 2.82	0.746
Basal E2(pg/mL)	42.96 ± 27.59	36.60 ± 15.78	41.09 ± 23.79	47.78 ± 39.07	0.651
Basal AFC	13 ± 4	13 ± 5	12 ± 6	13 ± 6	0.240
Protocol					0.147
Long agonist	26(68.4%)	21 (65.6%)	25(69.4%)	20 (55.6%)	
Short agonist	1(2.6%)	4 (12.5%)	4(11.1%)	6 (16.7%)	
Antagonist	11(29.0%)	7 (21.9%)	7(19.5%)	10 (27.7%)	
Insemination					0.706
IVF	23(60.5%)	21(65.6%)	22(61.1%)	26(72.2%)	
ICSI	15(39.5%)	11(34.4%)	14(38.9%)	10(27.8%)	
Total gonadotrophin used (IU)	1971.7 ± 797.8	2217.2 ± 791.2	2467.4 ± 1129.9	2406.9 ± 1033.3	0.090
Duration of hormonal stimulation(d)	9.5 ± 1.2	9.9 ± 1.2	10.1 ± 1.5	10.1 ± 1.2	0.055
Endometrial thickness on HCG day(cm)	1.03 ± 0.18	0.97 ± 0.17	1.01 ± 0.17	1.03 ± 0.19	0.446
Peak E_2_ on HCG day (pg/mL)	4071.4 ± 2582.5	4154.5 ± 2283.6	3291.3 ± 2135.0	2960.5 ± 1985.9	0.060
Number of transferred embryos	2.0 ± 0.6	2.0 ± 0.6	2.0 ± 0.5	2.0 ± 0.5	0.527

*AFC* Antral follicular count, *2PN* Two pronuclear zygote, *ICSI* Intracytoplasmic sperm injection, *IVF* In vitro fertilization

Data are presented as mean ± SD or n (%)

All patients were monitored for complications following the Pipelle biopsy. The endometrial injury was done successfully in all attempted subjects. There were no reports of complications including serious pain, pelvic infection, or excessive bleeding following the procedure of endometrial aspiration.

For our primary and secondary outcomes, we first compared the overall effect of endometrial injury regardless of menstrual cycle phase vs. non-injury treatment (Table 3). All the patients underwent embryo transfer. Mean implantation rates were similar between injury group and non-injury group. There were no significant differences in biochemical pregnancy, clinical pregnancy, miscarriage rate, ectopic pregnancy, multiple pregnancies, live birth rate between injury group and non-injury group.

**Table 3:** Pregnancy outcomes of patients undergoing endometrial injury and non-injury groups

Variable	Injury	Non-injury	*P* value
	(*n* = 70)	(*n* = 72)	
Number of transferred embryos	2.0 ± 0.6	2.0 ± 0.5	0.524
Implantation rate	40/147(27.2%)	39/146(26.7%)	1.000
Biochemical pregnancy rate	38/70(54.3%)	38/72(52.8%)	0.868
Clinical pregnancy rate	29/70(41.4%)	30/72(41.7%)	1.000
Miscarriage rate	1/29(3.4%)	1/30(3.3%)	1.000
Ectopic pregnancy rate	1/29(3.6%)	1/30(3.5%)	0.447
Multiple pregnancy rate	10/29(34.5%)	9/30(30 .0%)	0.785
Live birth rate	25/70(35.7%)	29/72(40.3%)	0.607

Data are presented as mean ± SD or n (%)

Subgroup analysis was performed by stratifying women into those undergoing injury in proliferative phase and luteal phase in Table 4. Mean implantation rates were similar among the four groups. There were also no significant differences in biochemical pregnancy rate, clinical pregnancy rate, miscarriage rate, ectopic pregnancy, multiple pregnancies or live birth rates among the four groups.

**Table 4:** Pregnancy outcomes of patients undergoing endometrial injury in proliferative phase, luteal phase and control groups

Variable	Group A	Group B	Group C	Group D	*P* value
	(*n* = 38)	(*n* = 32)	(*n *= 36)	(*n* = 36)	
Implantation rate	24/80(30.0%)	16/67(23.9%)	19/74(25.7%)	20/72 (27.8%)	0.853
Biochemical pregnancy rate	20/38(52.6%)	18/32(56.3%)	19/36(52.8%)	19/36 (52.8%)	0.989
Clinical pregnancy rate	16/38(42.1%)	13/32(40.6%)	16/36(44.4%)	14/36 (38.9%)	0.970
Miscarriage rate	1/16(6.3%)	0/13(0.0%)	0/16 (0.0%)	1/14(7.1%)	0.568
Ectopic pregnancy rate	0/16(0.0%)	1/13(7.7%)	0/16 (0.0%)	0/14(0.0%)	0.308
Multiple pregnancy rate	7/16(38.9%)	3/13(23.1%)	3/16 (18.8%)	6/14(42.9%)	0.401
Live birth rate	15/38(39.5%)	10/32(31.3%)	16/36(44.4%)	13/36 (36.1%)	0.719

Data are presented as mean ± SD or n (%)

## Discussion

In the present study, our aim was to assess whether there is a difference between proliferative phase and luteal phase endometrial injury on IVF outcomes. To the best of our knowledge, there has been no report comparing these two phases before. We found that there is no difference between injury in the proliferative phase or luteal phase in terms of IVF outcomes. Moreover, neither phase-specific endometrial injury in the preceding cycle resulted in significant improvement in implantation, clinical pregnancy, or live birth rates compared with control non-injury group among unselected subfertile women undergoing IVF.

The effect of endometrial injury on IVF cycle outcomes has been studied for over a decade, with studies reporting largely conflicting data. It was reported that the endometrial injury performed on the day of oocyte retrieval in the transfer cycle resulted in detrimental effects on the outcomes of IVF [14]. However, different results on IVF cycle outcomes have been obtained with endometrial injury performed in the cycle prior to the embryo transfer cycle. Several studies have reported that endometrial injury in the cycle prior to ovarian stimulation in IVF improved clinical pregnancy and/or live birth rates. Barash et al. demonstrated that repeated endometrial biopsies by a Pipelle catheter during both proliferative and luteal phase of the cycle preceding IVF doubled clinical pregnancy and live birth rates in women with history of one or more cycles of IVF failure [9]. Raziel et al. showed that endometrial biopsy by a Pipelle catheter in luteal phase benefited ICSI patients with repeated implantation failure [13]. Likewise, Zhou et al. reported that endometrial biopsy by a catheter performed under the guidance of B-ultrasound in proliferative phase of the IVF cycle improved implantation, clinical pregnancy and live birth rates in patients with irregular echoes diagnosed by ultrasound before IVF [10]. Moreover, a meta-analysis showed that endometrial injury (biopsy/scratch or hysteroscopy) performed in proliferative and/or luteal phase was 70% more likely to result in clinical pregnancy compared with no intervention, especially in unexplained RIF patients [11].It was reported that endometrial injury using a single-time curette biopsy in the proliferative phase of the preceding menstrual cycle improved the pregnancy outcome of RIF patients with uncompromised ovarian reserve [20].Recently it was suggested that hysteroscopy with local injury (with grasping forceps or scissors) to the luteal phase endometrium prior to ovarian stimulation for IVF/ICSI can improve implantation and pregnancy rates in RIF patients [21].

In contrast with the afore mentioned studies, our study demonstrated that endometrial injury performed in the preceding cycle did not improve the outcomes of IVF. These results are consistent with other studies. A study of oocyte donation recipients demonstrated no increase in implantation, clinical pregnancy or live birth rates in patients who underwent a single endometrial injury by a pipelle catheter performed in luteal phase compared as compared with controls. [7].A more recent study of patients with a history of at least one IVF failure undergoing a single luteal phase endometrial biopsy by a Pipelle catheter, demonstrated that biopsy in the cycle preceding IVF did not increase implantation, clinical pregnancy, or live birth rates compared with biopsy performed more than one cycle before IVF [18]. Recently, a systematic literature review about endometrial injury for RIF concluded that evidence is lacking for endometrial injury to be used in women with RIF undergoing ART [22].

It is probable that the heterogeneity of different subjects, different cycles, different phase of endometrial injury, the degree of injury, as well as the timing and number of endometrial biopsies contribute to the variable effects on IVF outcomes reported.

While most of the studies to date focused on specific subpopulations of infertile women such as those with RIF, there are few studies on the effect of endometrial injury in unselected subfertile women. In the present study, unselected subfertile women were recruited. We found that endometrial injury in unselected subfertile women results in no improvement to implantation, clinical pregnancy and/or live birth rates. This is consistent with other studies. A study which included 300 unselected subfertile women undergoing IVF/ICSI treatment who had endometrial aspiration using a Pipelle catheter in mid-luteal phase, concluded that endometrial injury in the preceding cycle did not result in improved ongoing pregnancy rate [5]. Moreover, a retrospective study recently reported that mechanical endometrial injury with Pipelle catheter in luteal phase did not improve implantation and pregnancy rates in a population of unselected subfertile women as compared to matched controls [23].

Proliferative phase and luteal phase are two important stages of the menstrual cycle, each with its own characteristics. Proliferative phase is histologically characterized by short glands, with the dense lining of the uterus and richly vascular. Luteal phase includes the ‘implantation window’, characterized by a large content of growth factors, cytokines and immune cells in the endometrium [24]. It is possible that endometrial injury performed in these two different phases may result in different outcomes of IVF cycles. However, in previous studies endometrial injury was performed either in proliferative phase or luteal phase or both, and no prior study compared the effect of endometrial injury between proliferative phase and luteal phase. Our data showed that endometrial injury in the preceding cycle in proliferative phase was comparable to luteal phase in terms of IVF outcomes, and that neither had significant benefit in terms of implantation, pregnancy and live birth rates as compared to non-injury controls. It is plausible that no difference was observed in outcomes between the two injury groups in our study because of the patient population of unselected subfertile women. Therefore, studies are warranted to examine the effect of injury in proliferative vs. luteal phase also in other populations such as RIF.

Several theories have been put forward to explain the mechanism of endometrial injury in improving implantation and pregnancy rates, including mechanical, inflammation, wound healing, and neoangiogenesis theories [25–30]. Local endometrial injury is thought to increase decidualization favorable to embryo implantation [25], and induce secretion of cytokines and growth factors facilitating decidualization and implantation [26, 27]. Alternatively, an inflammatory response may be triggered by endometrial injury with up-regulation of cytokines, adhesion molecules, and growth factors [28, 29]. Moreover, endometrial injury up-regulates endometrial gene expression profile related to endometrial receptivity [10, 29, 30]. It would be interesting to investigate whether endometrial injury in proliferative vs. luteal phase results in differences in molecular and/or inflammatory signatures in the endometrium in the subsequent cycle.

Strengths of our study were its randomized nature and the inclusion of a placebo group which had a sham procedure in the cervix, thus blinding patients to the intervention. The main limitation of our study was its sample size, and it would be beneficial to include more patients in each group.

## Conclusions

In conclusion, endometrial injury in the proliferative phase or luteal phase in the cycle preceding IVF of unselected subfertile women does not increase implantation, clinical pregnancy, or live birth rates compared with noinjury to the endometrium. Furthermore, there is no significant difference in IVF outcomes between endometrial injury in the proliferative phase or luteal phase. Studies with larger number of subjects and other infertility populations are needed. The different mechanism underlying the effects of injury between proliferative phase and luteal phase also need to be investigated.

## Abbreviations

2PN: Two pronuclear zygote
AFC: Average follicle count
ICSI: Intracytoplasmic sperm injection
IVF: In vitro fertilization
PCOS: Polycystic ovarian syndrome
